# Retrospective Evaluation of Childhood Germ Cell Tumors: A Single-Center Experience

**DOI:** 10.3390/children13010036

**Published:** 2025-12-26

**Authors:** Arzu Selamioglu, İbrahim Kartal, Oğuz Salih Dincer, Burak Tander, Murat Elli, Sükriye Bilge Gürsel, Sabri Acar, Ayhan Dagdemir

**Affiliations:** 1 Division of Pediatric Metabolic Diseases, Bağcılar Training and Research Hospital, Istanbul 34214, Turkey; 2 Division of Pediatric Hematology and Oncology, Faculty of Medicine, Ondokuz Mayıs University, Samsun 55270, Turkey; ibrahim_kartal28@hotmail.com (İ.K.); oguzssdincer@gmail.com (O.S.D.); ayhandagdemir@gmail.com (A.D.); 3 Department of Pediatric Surgery, School of Medicine, Acıbadem University, Istanbul 34750, Turkey; burak.tander@acibadem.edu.tr; 4 Division of Pediatric Hematology and Oncology, School of Medicine, Istanbul Medipol University, Istanbul 34810, Turkey; melli@medipol.edu.tr; 5 Department of Radiation Oncology, Faculty of Medicine, Ondokuz Mayıs University, Samsun 55270, Turkey; bgursel@omu.edu.tr; 6 Department of Pediatrics, Medicana Hospital, Samsun 55080, Turkey; arzuceyln@gmail.com

**Keywords:** children, germ cell tumors, sacrococcygeal region, mature cystic teratomas, endodermal sinus tumors

## Abstract

**Highlights:**

**What are the main findings?**

- Gonadal germ cell tumors were more frequent than extragonadal tumors, with the sacrococcygeal region representing the most common primary site in early childhood.
- High overall and malignant survival rates were achieved using risk-adapted multimodal treatment, despite heterogeneity in tumor site, histology, and disease stage.

**What are the implications of the main findings?**

- Increased clinical awareness of typical presenting symptoms, combined with routine use of ultrasonoraphy and AFP testing, may facilitate earlier diagnosis and reduce advanced-stage presentation.
- Early referral to specialized pediatric oncology centers and complete surgical resection are essential to achieving high survival rates while minimizing treatment intensity and long-term morbidity.

**Abstract:**

Background: Germ cell tumors are benign or malignant tumors that originate from the human embryo’s primordial germ cells. This study aims to conduct a retrospective analysis of germ cell tumors followed up at our institution, including their epidemiological data, treatment, and prognosis. Patients and Methods: Ninety-three cases were included and retrospectively evaluated for socio-demographic features, clinical data, presenting symptoms, histopathological findings, localization, staging, treatment protocol, and survival analysis. Results: Patients were diagnosed between 10 days and 17 years 10 months (median 27.2 months); 37 (40.7%) were male, 54 (59.3%) female. The tumors were located in the sacrococcygeal region (33.3%), ovaries (26.8%), testes (25.8%), abdomen (7.5%), CNS (2.1%), liver, adrenal gland, anterior mediastinum, and spine. Thirty-nine lesions were benign, and 54 were malignant. Mature cystic teratomas (40.8%), endodermal sinus tumors (28.0%), mixed germ cell tumors (12.9%), immature teratomas (9.7%), germinoma (6.5%), gonadoblastoma (1.1%), and choriocarcinoma (1.1%) were the different types of histology. We observed metastases in 17 malignant cases, with the lungs being the most commonly affected (10.7%). Stages I, II, III, and IV included 16, 17, 11, and 10 cases, respectively. Survival rates for all cases were 95.8%, and for malignant tumors, they were 92.7%. For malignant cases, the event-free survival rate was 84.2%. Conclusions: The findings provide comprehensive epidemiological and clinical data on germ cell tumors, enhancing understanding of their distribution, treatment outcomes, and prognosis. The high survival rates observed highlight the effectiveness of current treatment protocols, as well as the importance of early diagnosis and appropriate management.

## 1. Introduction

Germ cell tumors (GCT) originate from the primordial germ cell, which normally develops into sperm and ova in the human embryo. They represent a heterogeneous group of tumors that can occur at any age and in both sexes. GCTs can arise in either the gonadal (ovarian or testicular) or extragonadal (mediastinal, sacrococcygeal, or other) sites. Adolescent males frequently observe embryonal carcinomas, teratomas, and mixed germ cell tumors (GCTs), while infants and young children most commonly experience teratomas and yolk sac tumors. Dysgerminomas and teratomas are the primary types seen in adolescent females [1,2]. Childhood GCTs most commonly occur in the sacrococcygeal region, followed by the ovaries. Germ cell tumors often spread to the lungs, liver, bones, bone marrow, the central nervous system (CNS), and lymph nodes (regional, paraaortic, prerenal, and supradiaphragmatic) [3]. GCTs account for 3% of malignancies in children under 15 years of age and almost 14% of malignancies in adolescents aged 15–19 years [4].

Most childhood germ cell tumors secrete alpha-fetoprotein (AFP) or human chorionic gonadotropin (hCG). These serum tumor markers are important for both diagnosis and patient monitoring as well as assessing preoperative serum levels, as a failure to return to normal levels could signify residual, recurrent, or progressive disease. Treatment strategies for germ cell tumors vary depending on the tumor’s histological type, tissue of origin, and disease stage. Surgical resection is essential for the treatment of many germ cell tumors. Benign teratoma, immature teratoma, and low-stage malignant germ cell tumors are treated with surgical excision. Chemotherapy has had positive outcomes in malignant germ cell tumors [5].

In this retrospective study, we analyzed the archived records of the patients with GCT to define the clinicopathologic characteristics of this rare group of tumors in the Turkish population. In addition, we assessed treatment outcomes and prognostic factors, and compared the results with the literature.

## 2. Methods

### 2.1. Patients and Methods

The medical records of patients diagnosed with primary ovarian, testicular, or extragonadal germ cell tumors GCTs and managed at the Pediatric Oncology Department of Ondokuz Mayıs University Faculty of Medicine between 1984 and 2016 were retrospectively reviewed. All eligible cases were identified through a comprehensive search of the institutional pediatric oncology archives, and patients with incomplete or unavailable medical documentation were excluded. For each patient, detailed epidemiological and clinical information was collected, including age, sex, risk factors, prenatal history, presenting manifestations, histopathological diagnosis, tumor site, disease stage, serum tumor marker levels, treatment protocols, radiotherapy exposure, relapse patterns, late toxicities, and cause of death. Event-free and overall survival outcomes were systematically evaluated. Overall survival was defined as the duration (in months) from the date of histological diagnosis to death or to the last recorded follow-up for surviving patients. Time to relapse was calculated as the interval between histological diagnosis and the first documented recurrence.

Serum AFP and β-hCG measurements were performed at the institutional biochemistry laboratories using automated chemiluminescent immunoassay analyzers. Although assay platforms were periodically updated over the study period as part of routine laboratory modernization, all instruments and protocols were validated and calibrated against international reference standards to ensure longitudinal comparability of results. Age-adjusted AFP reference intervals published by Ishiguro and Tsuchida (1994) were used exclusively to define normal ranges [6].

Tumors were categorized as gonadal or extragonadal GCTs. Histopathological subtypes were classified as germinoma, teratoma, embryonal carcinoma, yolk sac tumor, choriocarcinoma, gonadoblastoma, mixed GCTs, or other rare variants [7], according to a modified version of Dehner’s classification. Mature teratomas composed entirely of fully differentiated tissues were designated as benign. Immature teratomas were identified by the presence of immature neuroectodermal elements. Those lacking malignant germ cell components were categorized as potentially malignant, reflecting their intermediate biological behavior, whereas tumors containing malignant elements—such as yolk sac tumor—were classified as malignant germ cell tumors. Immature teratomas were graded according to standard pediatric GCT pathology criteria. When malignant somatic components were present within a teratoma, these were interpreted as somatic-type malignant transformation and classified under malignant GCTs rather than as primary sarcomas.

Disease staging was performed using the unified POG/CCG system widely applied in pediatric extracranial GCTs. Stage I comprised completely resected, localized disease, including sacrococcygeal tumors treated with coccygectomy with negative surgical margins and no metastases. Stage II included tumors with positive or close margins or microscopic residual disease, in the absence of metastatic spread. Stage III encompassed macroscopic residual tumor or biopsy-only cases, irrespective of regional lymph node involvement. Stage IV included all patients with distant metastases [8,9]. Given the lengthy study period, all staging assignments were reassessed retrospectively through a review of operative reports, pathology records, imaging findings, and tumor marker kinetics to ensure strict adherence to contemporary POG/CCG criteria. Cases recorded using older staging terminology were reclassified according to current definitions for consistency.

In cases where histopathological confirmation was not available, a standardized approach was followed. Patients were clinically diagnosed based on tumor location, radiological characteristics, and markedly elevated AFP and β-hCG levels. These patients were included in the overall cohort description and survival analyses to preserve the integrity of longitudinal outcome evaluations; however, they were excluded from all subgroup analyses that required histology-based classification. Similarly, patients lacking AFP or β-hCG measurements were retained in survival analyses—because biomarker levels were not used as covariates—but omitted from analyses specifically examining associations between biomarker elevation and clinical outcomes.

### 2.2. Statistical Analysis

We used IBM SPSS Statistics version 25 for Windows for our statistical analysis. We prepared frequency tables for categorical variables and calculated descriptive statistics (mean, standard deviation, median, minimum, and maximum) for numerical variables. We assessed the significance levels for categorical comparisons between groups using cross-tabulations with the chi-square test. We used Mann–Whitney and Kruskal–Wallis test statistics for numerical comparisons for independent groups that did not meet the normal distribution assumption. We used the Kaplan–Meier test statistic for the survival analysis. Survival analysis was performed using the Kaplan–Meier method, and differences between groups were evaluated using the log-rank test. A Cox proportional hazards regression model was applied to assess the independent prognostic effects of clinical and laboratory parameters (diagnosis subtype, sex, stage, metastasis, AFP, hCG) on overall survival. A *p*-value less than 0.05 was considered statistically significant.

This study was reviewed and approved by the Ethics Committee of Ondokuz Mayıs University. Informed consent to participate in the study was obtained from the parents or legal guardians of all participants.

## 3. Results

### 3.1. Patients’ Characteristics

This study identified 93 cases of germ cell tumors at the Pediatric Oncology Department of Ondokuz Mayıs University Faculty of Medicine from 1984 to 2016. The age at the time of diagnosis ranged from 10 days to 17 years and 10 months (mean: 67.6 ± 72.01, median: 27.2 months). 37 (40.66%) were male, and 54 (59.34%) were female. Female patients were significantly more frequent than male patients (*p* = 0.002). The cases were divided into age groups at the time of diagnosis, revealing that out of 46 patients diagnosed between 1–60 months, 24 (52.1%) were male. The 37 patients older than 61 months included 11 males (29.7%). Among cases aged between 61 and 156 months, females predominated significantly (*p* = 0.001). All 10 neonatal patients (10.8%) presented with histologically benign tumors located in the sacrococcygeal region. During the antenatal period, we detected four cases (40%).

A person with 45,X/46,XY mixed gonadal dysgenesis, another person with 45,X0/46,XX mosaic hermaphroditism, and two people who had testes that had not fully descended were all at risk for germ cell tumors. The duration from presentation to diagnosis was found to range from 1 to 77 days (mean: 17.47 ± 14.99, median: 14 days). We observed that 25 patients (26.8%) presented with hip swelling, 23 patients (24.7%) with testicular swelling, 19 patients (20.4%) with abdominal pain, and 11 patients (11.8%) with abdominal swelling. In total, 62 patients (66.6%) sought medical attention due to mass-related complaints. In patients with tumors located in the CNS, one patient presented with seizures, and another experienced excessive thirst and headache. A physical examination revealed a mass in one patient with a persistent fever, who had tumors in the testicle. The other patients presented with testicle swelling.

Patients with tumors located in the ovary presented with abdominal pain in 17 cases (68.0%). Among cases with ovarian tumors, over torsion was present in 9 cases (36.0%) at the time of diagnosis. One patient with a tumor located in the sacrococcygeal region complained of being unable to walk, while the remaining cases complained of being mass related. The diagnosis of a patient with a tumor in the anterior mediastinum revealed the presence of superior vena cava syndrome. The distribution of cases based on the primary tumor location revealed that 31 cases (33.3%) were in the sacrococcygeal region, 25 cases (26.8%) were in the ovary, 24 cases (25.8%) were in the testicle, 7 cases (7.5%) were in the abdomen, and 2 cases (2.1%) were in the CNS. The study revealed that extragonadal tumors were present in 44 cases (47.3%), while gonadal tumors were present in 49 cases (52.7%). Among extragonadal tumors, 31 (70.4%) were female cases. While extragonadal tumors were more common in females, there was no significant difference in genders among the gonadal tumors (*p* > 0.05). Out of the 31 cases in the sacrococcygeal region, 27 (87%) were female. The predominance of sacrococcygeal tumors in females was statistically significant (*p* = 0.001).

**Figure 1:**
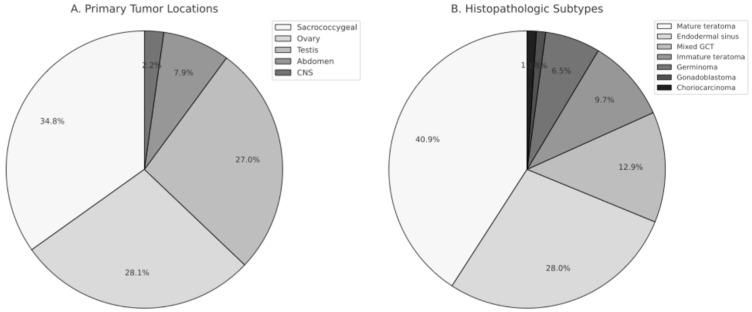
Distribution of Primary Tumor Locations and Histopathologic Subtypes in Pediatric Germ Cell Tumors. (**A**) Primary tumor locations, including sacrococcygeal region, ovary, testis, abdomen, and central nervous system (CNS). (**B**) Histopathologic subtypes of germ cell tumors, demonstrating the proportional distribution of mature teratoma, endodermal sinus tumor, mixed germ cell tumor, immature teratoma, germinoma, gonadoblastoma, and choriocarcinoma.

When evaluating the age at diagnosis based on the primary tumor location, it was observed that cases with sacrococcygeal tumors were diagnosed at ages ranging from 0.3 to 65.7 months (mean: 11 ± 15.3, median: 2.4 months), cases with testicular tumors were diagnosed at ages ranging from 2.6 to 209.1 months (mean: 68.6 ± 84.1, median: 23.6 months), and cases with ovarian tumors were diagnosed at ages ranging from 1.4 to 215 months (mean: 119 ± 50.3, median: 122.4 months). It was statistically significant that cases with sacrococcygeal tumors were younger at the time of diagnosis compared to other cases (*p* = 0.001). Cases with ovarian tumors were older at the time of diagnosis compared to cases with testicular tumors (*p* = 0.028). The distribution of tumor locations and histopathologic subtypes is shown in Figure 1.

### 3.2. Histological Diagnosis

Ninety-one cases were histologically confirmed, while histopathological evaluation was not available for two cases, both of whom were diagnosed before the year 2000. One presented with a sacrococcygeal mass, liver metastasis, and markedly elevated AFP levels, consistent with a presumed endodermal sinus tumor. The other had a third-ventricular/aqueductal tumor with markedly elevated AFP levels in serum and cerebrospinal fluid. These clinically diagnosed cases were included in the overall cohort analyses but excluded from all histopathology-based subgroup comparisons. Patients with missing AFP or β-hCG values were retained in survival analyses, as biomarker levels were not used as covariates, but were excluded from analyses examining associations between biomarker elevation and clinical outcomes. The distribution of histopathological diagnoses by primary tumor site is summarized in Table 1. A total of 39 cases (42%) were determined to have a benign origin, while 54 cases (58%) were found to be malignant. Of the cases with sacrococcygeal localization, 16 (51.6%) were benign, and 15 (48.4%) were malignant. In ovarian tumors, 18 cases (72%) were benign, and 7 cases (28%) were malignant. Among testicular tumors, 3 cases (12.5%) were benign, and 21 cases (87.5%) were malignant. Endodermal sinus tumor was the most frequently observed diagnosis in the malignant group, accounting for 28% of cases. Mature cystic teratoma was identified in 38 cases (40.8%), while mixed germ cell tumors were present in 12 cases (12.9%) and immature teratoma in 9 cases (9.68%). Additionally, germinoma, gonadoblastoma, and choriocarcinoma were identified in 6, 1, and 1 cases, respectively (6.45%, 1.08%, and 1.08%, respectively). Among the cases with mixed germ cell tumors, three had teratoma and endodermal sinus tumor, one had anaplastic seminoma and embryonal carcinoma, two had teratoma, embryonal carcinoma, and endodermal sinus tumor, one had endodermal sinus tumor and adenocarcinoma, and one had teratocarcinoma.

The patients diagnosed with mature teratoma ranged in age from 0.3 to 215 months (mean: 58.77 ± 66.82, median: 21.25 months). Those with endodermal sinus tumors were diagnosed at an age ranging from 2.4 to 208.7 months (mean: 41.76 ± 60.9, median: 21.2 months), and patients with germinomas were diagnosed at an age ranging from 110.8 to 115 months (mean: 157.9 ± 30.8, median: 171 months). The patient with gonadoblastoma was diagnosed at 178 months. Patients with germinoma were diagnosed at a significantly older age compared to other germ cell tumors (*p* < 0.05). Patients with mixed germ cell tumors were older than those with mature teratoma (*p* < 0.05). Table 2 summarizes the distribution of demographic characteristics, tumor features, and outcome measures according to age at diagnosis. The follow-up period ranged from 0.2 to 254 months (mean: 62.83 ± 51.97, median: 53.16 months).

Pre-treatment serum AFP levels were evaluated in 87 patients (93.5%), whose ages ranged from the neonatal period to late adolescence. Among these patients, 51 (58.6%) had age-appropriate normal AFP concentrations, while 36 (41.4%)—predominantly infants and young children—showed elevated levels. AFP values ranged widely from 0.4 to 130,000 ng/mL, and 15 patients older than 1 month had markedly elevated levels exceeding 10,000 ng/mL. Among patients with endodermal sinus tumors (median age: 21.2 months), AFP ranged from 3.5 to 130,000 ng/mL (mean: 20,539.5 ± 35,582.1), and 84% had elevated AFP levels. In mixed germ cell tumor cases (median age: 33 months), 81.8% demonstrated AFP elevation. Pre-treatment serum β-hCG levels were assessed in 85 patients (91.3%), spanning ages from early childhood to adolescence. β-hCG concentrations ranged from 0.1 to 2,057,360 mIU/mL, with 74 patients (87%) exhibiting normal values and 11 patients (13%)—mostly older children—showing elevated levels. Elevated β-hCG was observed in four patients with endodermal sinus tumors (median age: 21 months), three with mixed germ cell tumors, two adolescents with germinomas, one adolescent with immature teratoma, and one adolescent with choriocarcinoma. The choriocarcinoma case, diagnosed at 14 years of age, showed a profoundly elevated β-hCG level of 2,057,360 mIU/mL.

Metastasis was observed in 17 patients (18.3%), with lung metastasis detected in 8 patients, liver metastasis in 4 patients, abdominal metastasis in 4 patients, and lymph node metastasis in 4 patients. One patient had lung and liver metastasis, and one had lung and abdominal metastasis. Metastasis was observed in 30.7% of endodermal sinus tumor cases. Among the 12 patients with mixed germ cell tumors, 5 (41.6%) had metastatic disease. Two patients with germinoma had metastasis. The patient with choriocarcinoma had lung metastasis. The lower rate of metastasis in patients with endodermal sinus tumors and immature teratomas was statistically significant (*p* < 0.05).

Sixteen patients (30%) were in stage I, 17 (31%) in stage II, 11 (20%) in stage III, and 10 (19%) in stage IV. Among the cases of mixed germ cell tumors, four were in stage I (33.3%), four were in stage II (33.3%), one was in stage I (8.3%), and three were in stage IV (25.1%); among the cases of endodermal sinus tumors, eight were in stage I (29.7%), six were in stage II (22.2%), six were in stage III (22.2%), and seven were in stage IV (25.9%); among the germinoma cases, one was in stage I (16.6%), four were in stage II (66.8%), and one was in stage III (16.6%). Table 3 shows the distribution of histopathologies in malignant cases by stage. Among the gonadal tumors, 12 cases were in stage I (42.8%), 9 were in stage II (32.1%), 4 were in stage III (14.3%), and 3 were in stage IV (10.7%). Among the extragonadal tumors, four (15.4%), 8 cases were stage II, eight (30.8%), 7 cases were stage III, seven (26.9%), and 7 cases were stage IV, and seven (26.9%) were stage IV. Regarding sacrococcygeal localization, two cases (13.3%) were in stage I, six cases (40%) were in stage II, two cases (13.3%) were in stage III, and five cases (33.3%) were in stage IV. No stage IV ovarian tumors were observed.

### 3.3. Treatment Characteristics

With the exception of 4 patients, all individuals underwent surgical interventions for both histopathological diagnosis and therapeutic purposes. In one case with pineal localization, diagnosis was established through biopsy.

Five (62.5%) patients underwent surgery after relapse. Among patients with metastasis, surgery targeting metastatic sites was performed in 6 cases (35.3%). Of the surgically treated patients, 79 (88.8%) underwent total resection. Among the cases with partial resection, 5 (50%) were in the sacrococcygeal region, 1 (10%) in the testicle, 1 (10%) in the liver, 1 (10%) in the abdomen, 1 (10%) in the anterior mediastinum, and 1 (10%) in the adrenal region. Among the 31 cases localized in the sacrococcygeal region, 26 (83.9%) underwent total surgery. Following surgery, 46 patients (51.7%) received chemotherapy, while 43 (48.3%) did not require additional therapy. Among immature teratoma cases, two did not receive chemotherapy, four received the BEP (bleomycin, etoposide, cisplatin) protocol, and three received VAC (vincristine, actinomycin-D, cyclophosphamide) protocols. Cases treated with VAC protocols exhibited rhabdomyosarcomatous differentiation in their histopathology. Three cases with endodermal sinus tumors did not receive chemotherapy, while 23 cases received BEP protocol. Cases with inadequate response or relapse received ICE (ifosfamide, carboplatin, etoposide), VIP (etoposide, ifosfamide, cisplatin), and JEB (carboplatin, etoposide, bleomycin) protocols. In one case with endodermal sinus tumor, after surgery, BEP and ICE protocols were administered, followed by high-dose chemotherapy with stem cell rescue due to persistent disease. All patients not receiving chemotherapy had stage I gonadal tumors that were completely resected.

Radiation therapy was administered to five patients (5.4%), including one with endodermal sinus tumor, one with germinoma, one with immature teratoma, and two with mixed germ cell tumors. Among these patients, three underwent radiation therapy targeting the primary tumor site, one for metastasis, and one for recurrent tumor. Palliative radiation therapy was applied to brain and bone metastases in one case. Of those who received radiation therapy, two were in stage IV, one in stage III, and two in stage II. All patients with CNS localization and two (6.5%) with sacrococcygeal localization underwent radiation therapy. Among those who received radiation therapy, two underwent total resection, one underwent partial resection, and surgery was not performed on the other two cases. No cases received radiation therapy after 2006.

Nine patients (9.7%) experienced relapse. All relapses occurred in malignant tumors. Among the nine patients who experienced relapse, the time to recurrence ranged from 5.0 to 16.6 months, with a mean of 10.4 ± 3.7 months and a median of 11.0 months. Relapse cases were predominantly within the 12–60 month age group (5/9, 55.5%), followed by 1–12 months (2/9, 22.2%) and 156–216 months (2/9, 22.2%). Histologically, five of the relapsed patients (55.5%) had endodermal sinus tumors, while the remaining four (44.4%) had mixed germ cell tumors. No relapses occurred in patients with mature or immature teratomas or other histological subtypes. Seven of the nine patients (77.8%) had extragonadal tumors, most commonly originating from the sacrococcygeal or abdominal regions. Only two relapse cases (22.2%) were gonadal in origin. Metastatic disease was present at diagnosis in five of the relapsed patients (55.5%), including lung, liver, abdominal, pelvic, and spinal involvement. Out of the 17 cases with metastasis, 5 (29.4%) experienced relapse. No significant relationship was found between relapse and metastatic disease (*p* > 0.05). Three of the nine patients (33.3%) who relapsed ultimately died, all of whom had metastatic extragonadal tumors. Among the relapse cases, six patients (66.7%) had undergone total resection and three (33.3%) partial resection. Relapse occurred across all stages, most commonly in stage III and stage IV disease; however, due to the very small number of relapse events, no statistically significant association was observed between relapse and age group, gender, tumor location, histological subtype, stage, or presence of metastasis (all *p* > 0.05).

Toxicity was observed in a total of nine cases (9.7%) during the follow-up, including ototoxicity in five cases, ototoxicity and nephrotoxicity in one case, diabetes insipidus in two cases, and fecal incontinence in one case. All cases with toxicity, including 4 with endodermal sinus tumors, 3 with mixed germ cell tumors, 1 with germinoma, and 1 with immature teratoma, had received chemotherapy.

### 3.4. Outcome

**Figure 2:**
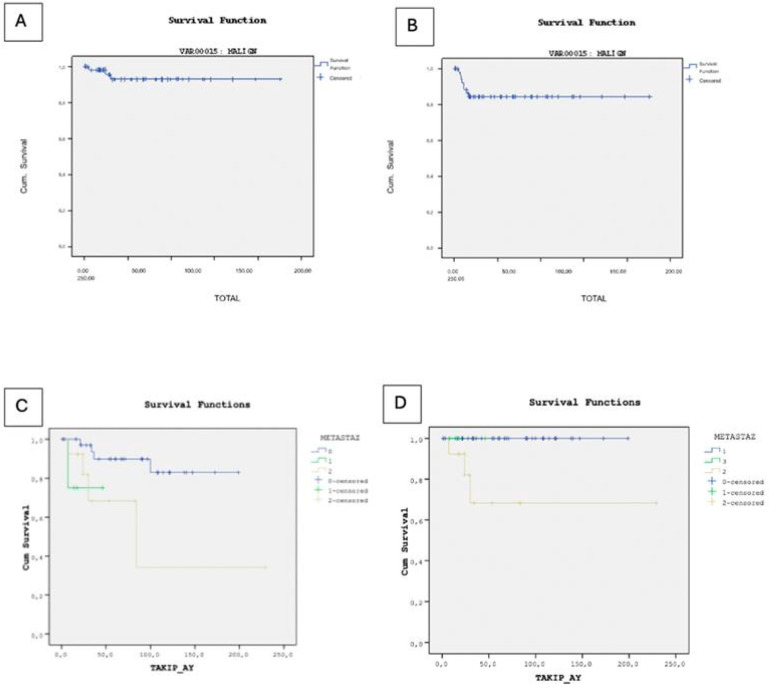
(**A**) Kaplan–Meier curve illustrating overall survival in malignant cases. (**B**) Kaplan–Meier curve illustrating event-free survival in malignant cases. (**C**) Kaplan–Meier analysis of overall survival stratified by metastatic status. (**D**) Kaplan–Meier analysis of event-free survival stratified by metastatic status.

In our study, 79 patients (84.9%) achieved remission, while 3 patients (3.2%) deceased, and 1 patient (1.1%) remained under active surveillance due to persistent disease at final follow-up. The deceased cases comprised two individuals with endodermal sinus tumors and one with a mixed germ cell tumor, all of whom experienced relapse and subsequently underwent surgical intervention. Specifically, two cases presented with lung metastases, and one with metastases in the abdomen, pelvis, and spine, all managed surgically. The overall survival rate for all germ cell tumors, including benign cases, was 95.8%. Among the malignant cases, the overall survival rate was 92.7%, with 9 cases experiencing recurrence, leading to an event-free survival rate of 84.2% (Figure 2).

In the Kaplan–Meier survival analysis for overall survival, only the presence of metastasis (*p* = 0.039) and tumor stage (*p* = 0.001) were statistically significant prognostic factors. Other clinical and laboratory variables—including diagnosis subtype (*p* = 0.777), sex (*p* = 0.052), AFP level (*p* = 0.291), β-hCG level (*p* = 0.371), tumor marker positivity (*p* = 0.449), and primary tumor site (*p* = 0.311) were not significantly associated with overall survival. However, in the multivariate Cox proportional hazards regression model for overall survival, none of the included clinical or laboratory variables, diagnosis subtype (HR = 1.017, *p* = 0.948), sex (HR ≈ 0, *p* = 0.904), tumor stage (HR = 1770.911, *p* = 0.865), presence of metastasis (HR = 4.060, *p* = 0.982), AFP level (HR = 0.829, *p* = 1.000), β-hCG level (HR = 6.559, *p* = 0.995), and tumor marker positivity (HR = 498.920, *p* = 0.990), showed statistically significant associations with overall survival. Although the overall model fit was statistically significant (χ^2^ = 15.234, *p* = 0.033), the individual covariates did not demonstrate independent prognostic value. The high standard errors and extreme hazard ratio estimates suggest model instability, likely due to the small number of events (n = 3) and a high censoring rate, which may have compromised the reliability of the Cox regression model.

Kaplan–Meier survival analysis revealed no statistically significant differences in event-free survival based on diagnosis subtype (*p* = 0.061), AFP level (*p* = 0.310), β-hCG level (*p* = 0.282), tumor marker positivity (*p* = 0.193), or primary tumor site (*p* > 0.05). However, the presence of metastasis was significantly associated with reduced event-free survival (*p* = 0.028). Although sex was borderline significant (*p* = 0.050), it is noteworthy that all three observed deaths occurred in female patients, and the limited number of events restricts the interpretability of this finding. In the multivariate Cox regression analysis for event-free survival, none of the clinical or laboratory variables were found to be statistically significant independent prognostic factors. Diagnosis subtype (HR = 1.071, *p* = 0.682), sex (HR = 0.248, *p* = 0.118), tumor stage (HR = 1.497, *p* = 0.531), presence of metastasis (HR = 1.405, *p* = 0.584), AFP level (HR = 0.510, *p* = 0.624), β-hCG level (HR = 0.294, *p* = 0.214), and tumor marker positivity (HR ≈ 0, *p* = 0.980) all showed no significant association with EFS. The small number of observed events (n = 9) may have limited the statistical power of the model.

## 4. Discussion

This study provides a comprehensive overview of our 32-year institutional experience with pediatric germ cell tumors, highlighting key epidemiological patterns, histopathological distributions, and treatment outcomes across a diverse spectrum of gonadal and extragonadal presentations. In our study, gonadal tumors were more prevalent than extragonadal tumors. Similarly, Harms et al. reported that 54.1% of the cases were gonadal tumors and 45.9% were extragonadal tumors [8]. Gonadal germ cell tumors are more common than extragonadal tumors because primordial germ cells normally migrate to the genital ridges during embryogenesis, resulting in the gonads harboring the highest concentration of germ cells. This anatomically and hormonally active microenvironment supports germ cell proliferation and increases the likelihood of malignant transformation. In contrast, extragonadal tumors arise from rare ectopic germ cells that fail to complete normal migration, making them inherently less frequent [9]. In the series published by Harms et al., 30.6% of tumors were benign, 17.6% were potentially malignant, and 51.8% were malignant; moreover, 10 patients (19.6%) had died and 4 patients (7.8%) were living with persistent disease at follow-up [8]. Although the anatomical distribution and histopathological profile in our cohort were highly consistent with those reported by Harms et al., our clinical outcomes were substantially more favorable, with markedly lower mortality and persistent disease rates. Considering that the Harms et al. study reflects clinical practice from the 1980s, this discrepancy likely reflects substantial improvements in diagnostic imaging, surgical techniques, chemotherapy protocols, and supportive care over the past decades.

The most common location of germ cell tumors in childhood is the sacrococcygeal region [10], and our findings were consistent with the literature, with 33.3% of all cases arising in this area. Yoshida et al. reported that among 289 sacrococcygeal tumors in Japan, mature teratomas were the predominant subtype (67.1%), followed by immature teratomas (16.3%) and endodermal sinus tumors (16.6%) [11]. Similarly, mature teratoma was the most frequent subtype in our sacrococcygeal cases. This alignment across geographically distinct populations suggests that the biological behavior and histogenetic pathways of sacrococcygeal GCTs are highly conserved. Yang et al. reported a comparable predominance of mature teratomas in ovarian GCTs, with 56.9% of tumors classified as mature teratomas and 37.2% as endodermal sinus tumors. In their series, abdominal pain and distension were common presenting symptoms, and ovarian torsion occurred in 15.3% of patients—findings that closely mirror our observations [12]. Similarly, studies by Schmidt et al. and Nguyen et al. demonstrated that endodermal sinus tumor is the most common malignant subtype in testicular GCTs, accounting for more than half of the cases in both series [13,14]. Our results showed a comparable distribution, with 54.1% of testicular tumors classified as endodermal sinus tumors. Taken together, these consistent patterns across sacrococcygeal, ovarian, and testicular tumors indicate that the histopathological profile of pediatric GCTs is remarkably stable across different populations and treatment eras. This concordance reinforces the generalizability of our findings and suggests that underlying biological mechanisms, rather than regional or temporal factors, primarily shape the distribution of tumor subtypes [9].

Rare cases of pediatric germ cell tumors have been reported in unusual sites such as the tongue, tonsil, liver, ileum, mesentery, eye, vulva, anorectal region, and retroperitoneum [10]. In our cohort, the distribution of tumor locations was consistent with the literature; however, no cases arose in these rare head and neck regions. Multiple histological subtypes may occur within a single tumor in approximately 25% of pediatric GCTs [3], and in our study, mixed germ cell tumors were identified in 12 out of 93 cases. This proportion closely aligns with the 12.7% rate reported by Schneider et al. in a large German cohort [15], supporting the notion that mixed histology represents a stable and biologically characteristic feature of pediatric GCTs. Regarding anatomical sites, 51.6% of sacrococcygeal tumors in our study were benign, whereas 72% of ovarian tumors were benign and 87.5% of testicular tumors were malignant. These patterns are consistent with the literature, including the findings of Vaysse et al., who reported that 77.3% of ovarian GCTs in their pediatric cohort were benign and 22.7% malignant [16]. The concordance between our results and published series suggests that the biological behavior of GCTs at different anatomical locations—particularly the high benignity rate in ovarian tumors and the predominance of malignant histology in testicular tumors—is reproducible across populations and likely reflects intrinsic differences in germ cell biology rather than institutional or regional variability.

The distribution of germ cell tumors by sex varied according to tumor location. Extragonadal tumors were more common in females (70.4%), whereas no significant sex difference was observed among gonadal tumors. The markedly higher prevalence of sacrococcygeal tumors in females (87%) in our study was statistically significant (*p* = 0.001). This sex-related pattern is consistent with the findings of Harms et al., who also reported a predominance of sacrococcygeal GCTs in females [17]. Most cases in our cohort (60.2%) were diagnosed before the age of 5, aligning with the results of Nguyen et al., who found that 78.6% of extracranial GCTs occurred in children younger than 5 years [14]. All neonatal cases in our cohort (10.8%) presented with benign sacrococcygeal tumors and exhibited a male-to-female ratio of 1:4. Similar observations were reported by Swamy et al., who noted the same 1:4 ratio in congenital sacrococcygeal teratomas, 10% of which demonstrated malignant histology [18]. These findings confirm several well-recognized epidemiological trends: (1) sacrococcygeal tumors show a strong female predominance, (2) the majority of pediatric GCTs present in early childhood, and (3) congenital sacrococcygeal teratomas are typically benign. During the antenatal period, we detected four cases (40%), underscoring the critical role of prenatal ultrasonography in the early identification of sacrococcygeal tumors. Fetal sacrococcygeal teratomas may be associated with serious complications, including high-output cardiac failure, hydrops fetalis, polyhydramnios, and intrauterine fetal demise, particularly in rapidly growing and hypervascular tumors. Current management relies primarily on close antenatal surveillance with serial ultrasonography and Doppler assessment. Although fetal interventions such as vascular ablation or cyst drainage have been described in selected high-risk cases, these procedures remain uncommon due to their associated risks. Nevertheless, available data suggest that fetal intervention may confer a survival benefit in a limited subset of patients who would otherwise be at high risk of fetal demise. In our cohort, all antenatally detected cases followed a benign course and did not require fetal intervention [19].

Germ cell tumors most commonly spread via lymphatic and hematogenous routes. Lymph node involvement is frequently observed in testicular and ovarian tumors, while distant metastases most often involve the lungs and liver [20]. In our cohort, 18.3% of patients had metastatic disease at diagnosis, a rate consistent with previous reports indicating distant metastasis in 15–30% of cases at presentation [21]. Among patients with metastatic disease, endodermal sinus tumor was the most common histological subtype, accounting for 8 of 17 cases, corresponding to a metastasis rate of 30.7% within this group. This finding is comparable to the study by Cornejo et al., who reported metastatic disease in 24.4% of patients with testicular endodermal sinus tumors [22]. In contrast, metastasis was significantly less frequent in patients with immature teratomas and selected other histological subtypes (*p* < 0.05). Regarding ovarian germ cell tumors, 14.3% of cases were stage I, 42.9% stage II, and 42.9% stage III at diagnosis, with no stage IV disease observed. Similar variability in stage distribution has been reported in the literature. Backer et al. reported predominantly early stage disease in pediatric ovarian GCTs [23], whereas Billmire et al. observed a higher proportion of advanced-stage tumors [24], likely reflecting differences in referral patterns and timing of diagnosis. Given that many pediatric germ cell tumors initially present with nonspecific yet detectable symptoms—such as painless scrotal or abdominal swelling, abdominal pain, increasing abdominal girth, or a palpable mass—early suspicion at the primary care level may facilitate timely referral to specialized centers. In this context, family physicians and primary care pediatricians play a crucial role in recognizing warning signs and initiating early diagnostic evaluation. Beyond clinical examination, first-line diagnostic tools available in primary care settings—particularly ultrasonography—are pivotal for the early detection of gonadal and abdominal masses. Ultrasonography is noninvasive, widely accessible, and highly sensitive for identifying testicular, ovarian, and abdominal tumors, enabling prompt differentiation between benign conditions and lesions requiring urgent specialist assessment. In addition, serum tumor markers, especially AFP and β-hCG, represent an important adjunct in the initial evaluation. Markedly elevated AFP and β-hCG levels, even in the absence of definitive imaging findings, should raise suspicion for malignant germ cell tumors and prompt expedited referral to tertiary care.

Germ cell tumors are rare pediatric cancers with excellent outcomes when treated according to well-established international guidelines. The introduction of platinum-based chemotherapy in the 1980s significantly improved the survival rates, with nearly 100% survival in stage I GCT. The first approach in the treatment of germ cell tumors is surgical resection [25]. Except for 4 of our cases, all underwent surgery for both histopathological diagnosis and treatment. The diagnosis in a case located in the pineal region was made by biopsy. Another case with a tumor located in the third ventricle and aqueduct was diagnosed by demonstrating high AFP levels in serum and cerebrospinal fluid. A patient with an abdominal mass was diagnosed by biopsy and lost to follow-up while undergoing treatment. Another case with a mass extending to the rectal region from the sacrococcygeal area was diagnosed by biopsy. In the study by Popadiuk et al., involving 95 pediatric germ cell tumor cases in Poland between 1998 and 2000, 88% of the patients were diagnosed histopathologically, while 12% were diagnosed through imaging and tumor markers [26]. Surgical treatment was performed after chemotherapy in 14 patients who had histopathological diagnoses by biopsy. Surgery was performed in 5 out of 9 cases (55.6%) with recurrence. Among patients with metastasis, 6 cases (35.3%) underwent surgery targeting the metastasis.

Following surgery, 51.7% of patients in our cohort received chemotherapy, whereas 48.3% did not require additional treatment. This distribution reflects a risk-adapted treatment approach, particularly in patients with completely resected, early stage gonadal tumors. Comparable variations in treatment strategies have been reported across different institutional series. In a large Japanese review by Suita et al., surgical intervention was performed in 116 of 117 pediatric GCT cases, with total excision achieved in 88 patients and incomplete resection in 28. Adjuvant chemotherapy was administered to 88 patients, high-dose chemotherapy with stem cell transplantation to one patient, and radiotherapy to 21 patients [27]. Similarly, Curto et al. reported data from an Italian cohort of 95 patients, in which only 32.6% underwent total surgical excision, predominantly comprising stage I testicular tumors and early stage ovarian dysgerminomas. In that series, postoperative chemotherapy was administered to 63 patients [28]. Differences in surgical completeness and chemotherapy utilization among studies likely reflect heterogeneity in tumor stage at diagnosis, histopathological distribution, and evolving treatment protocols over time. In our cohort, the selective use of chemotherapy underscores the importance of complete surgical resection and accurate staging in minimizing treatment intensity without compromising oncologic outcomes.

Recurrence was observed in 19.3% of sacrococcygeal tumors, 14.2% of abdominal tumors, and 4.2% of testicular tumors. The significantly higher recurrence rate observed in sacrococcygeal tumors underscores the importance of complete resection, including coccygectomy, as part of primary surgical management (*p* = 0.001). The associations between relapse and various clinical parameters are summarized in Table 4. Comparable findings have been reported in the literature. In a multicenter study conducted by Derikx et al. in the Netherlands, the recurrence rate within three years after surgery for sacrococcygeal germ cell tumors was 11%, with partial resection and immature or malignant histology identified as the most significant risk factors for recurrence [29]. Similarly, Yoneda et al. reported late recurrence rates of 7.8% in mature teratomas and 6% in immature teratomas of the sacrococcygeal region in a large Japanese cohort [30]. Taken together, these findings emphasize that meticulous surgical technique and complete excision at initial surgery are critical determinants of long-term disease control in sacrococcygeal germ cell tumors.

Long-term survival rates for pediatric germ cell tumors have improved substantially over recent decades with advances in multimodal treatment strategies. In a study by Vaysse et al. investigating pediatric ovarian germ cell tumors, recurrence and mortality rates were both reported as 1.3% [16]. Similarly, Backer et al., in a cohort of 66 pediatric patients with ovarian germ cell tumors, observed a recurrence rate of 4.5% and a mortality rate of 3% [23]. In the present study, the overall survival rate for all germ cell tumors was 95.8%, while survival among malignant cases reached 92.7%. Although we evaluated prognostic factors using log-rank testing, the very low number of deaths and relapses limited statistical power. As a result, the absence of statistically significant associations should be interpreted with caution, and does not exclude the clinical relevance of these variables. In a study by Frazier et al. involving 519 germ cell tumor cases from 1985 to 2009, stage IV disease, age ≥ 11 years, and tumor location were found to negatively affect prognosis [31]. In the study by Curto et al., it was reported that patients with AFP levels above 10,000 ng/mL had lower survival rates compared to those with lower AFP levels [28]. Although AFP levels ≥ 10,000 were associated with poor prognosis, cases with pure endodermal sinus tumors showed better outcomes. In this context, AFP should be regarded primarily as a sensitive diagnostic and monitoring biomarker rather than a standalone prognostic indicator. In our cohort, elevated AFP levels at diagnosis did not independently improve prognosis; rather, AFP functioned as a highly sensitive biomarker that facilitated the early recognition of malignant germ cell tumors. This early biochemical signal often prompted timely referral from primary care settings to specialized pediatric oncology units, thereby accelerating diagnostic imaging, surgical planning, and treatment initiation. Such early steps may indirectly contribute to improved outcomes, particularly in yolk sac tumors where delays in diagnosis are strongly associated with advanced-stage presentation and relapse. It is important to note that previous studies have reported inconsistent associations between AFP elevation and survival, highlighting that AFP should be interpreted primarily as a diagnostic and monitoring tool rather than as a standalone prognostic indicator. Given its wide availability and low cost, AFP testing represents an important early warning marker that can be ordered even by family physicians, serving as a critical trigger for rapid referral and optimized management of pediatric germ cell tumors [31]

Although therapeutic approaches for pediatric germ cell tumors have evolved substantially over the past three decades, a meaningful comparison of outcomes across treatment eras could not be performed in our cohort. Only five patients were diagnosed prior to 2000, and none of them experienced relapse or mortality. The extremely small number of early era cases limits the statistical power required for an era-stratified assessment of survival or relapse. Consequently, although we attempted an exploratory comparison, Fisher’s exact test demonstrated no statistically significant differences between the 1984–1999 and 2000–2016 periods (*p* > 0.05). This absence of significance likely reflects inadequate sample size rather than a true lack of clinical improvement over time. Therefore, the interpretation of temporal trends should be made with caution, and larger multicenter datasets would be required to meaningfully assess changes in outcomes related to advancements in diagnostic methods, surgical techniques, and chemotherapeutic protocols.

Another limitation of our study is that two patients—both from the pre-2000 era—lacked histopathological confirmation and were diagnosed clinically based on AFP elevation and tumor characteristics. Although they were included in the overall cohort, the absence of definitive histology prevented their inclusion in subgroup analyses. Additionally, AFP and β-hCG values were not available for all patients, which may modestly limit the precision of biomarker-related interpretations.

In this large retrospective study, we described patients with GCTs and provided information on clinical features, treatments, and outcomes. Detailed comparison of outcomes in pediatric GCTs treated with multi-modality regimens across various trials has been challenging due to their inherent heterogeneity, relative rarity, and lack of uniform risk stratification among major study groups. Although our study was not based on a population-based tumor registry, it may still provide representative data of germ cell tumors in our state, given that the cases were collected from a tertiary referral center. In the future, detailed multi-institutional studies focusing on community prevalence, etiology, and molecular biology, along with the global exchange of data, could enhance our understanding of this rare group of GCTs.

## Figures and Tables

**Table 1:** Distribution of histopathology by primary localization of tumor region.

Histopathology	Sacrococcygeal *n* (%)	Abdomen *n* (%)	Testicle *n* (%)	Ovary *n* (%)	CNS *n* (%)	Others *n* (%)
Endodermal sinus tumor	8 (25.8)	3 (42.8)	13 (54.1)	0 (0)	1 (50)	1 (25)
Mature teratoma	16 (51.6)	1 (14.2)	3 (12.5)	17 (68)	0 (0)	1 (25)
Immature teratoma	2 (6.4)	1 (14.2)	2 (8.3)	3 (12)	0 (0)	1 (25)
Mixed germ cell tumor	5 (16.1)	2 (28.5)	4 (16.6)	1 (4)	0 (0)	0 (0)
Germinoma	0 (0)	0 (0)	2 (8.3)	3 (12)	1 (50)	0 (0)
Gonadoblastoma	0 (0)	0 (0)	0 (0)	1 (4)	0 (0)	0 (0)
Choriocarcinoma	0 (0)	0 (0)	0 (0)	0 (0)	0 (0)	1 (25)

CNS: central nervous system.

**Table 2:** Distribution of demographic, tumor characteristics, and outcome indicators by diagnosis age group.

Category	0–1 mn *n* (%)	2–12 mn *n* (%)	13–60 mn *n* (%)	61–156 mn *n* (%)	157–216 mn *n* (%)
Male	2 (5.4)	9 (24.3)	15 (40.5)	1 (2.8)	10 (27)
Female	8 (14.3)	9 (16.1)	13 (23.2)	19 (33.9)	7 (12.5)
Endodermal sinus tumor	0 (0)	6 (23)	16 (61.5)	1 (3.8)	3 (11.5)
Mature teratoma	10 (26.3)	7 (18.4)	6 (15.7)	12 (31.5)	3 (7.9)
Immature teratoma	0 (0)	3 (33.3)	1 (11.1)	2 (22.2)	3 (33.3)
Mixed germ cell tumor	0 (0)	1 (8.3)	5 (41.6)	3 (25)	3 (25)
Germinoma	0 (0)	0 (0)	0 (0)	2 (33.3)	4 (66.6)
Gonadoblastoma	0 (0)	0 (0)	0 (0)	0 (0)	1 (100)
Choriocarcinoma	0 (0)	1 (100)	0 (0)	0 (0)	0 (0)
Benign	10 (25.6)	7 (18)	6 (15.3)	12 (30.8)	4 (10.3)
Malign	0 (0)	11 (20.4)	22 (40.7)	8 (14.8)	13 (24.1)
Gonadal	0 (0)	7 (14.3)	14 (28.6)	15 (30.6)	13 (26.5)
Extragonadal	10 (22.7)	11 (25)	14 (31.8)	5 (11.4)	4 (9.1)
Metastasis+	0 (0)	2 (20)	3 (30)	0 (0)	5 (50)
Metastasis−	10 (12)	16 (19.3)	25 (30.1)	20 (24.1)	12 (14.5)
Relapse+	0 (0)	2 (22.2)	5 (55.6)	0 (0)	2 (22.2)
Relapse−	10 (11.9)	16 (19.1)	23 (27.4)	20 (23.8)	15 (17.8)
Exitus	0 (0)	1 (33.3)	2 (66.7)	0 (0)	0 (0)

months: mn.

**Table 3:** Distribution of Histopathologies in Malignant Cases by Stage.

Histopathology	Stage I *n* (%)	Stage II *n* (%)	Stage III *n* (%)	Stage IV *n* (%)	Total *n*
Endodermal sinus tumor	8 (30.7)	6 (23)	6 (23)	6 (23)	26
Immature teratoma	3 (33.3)	3 (33.3)	3 (33.3)	0 (0)	9
Mixed germ cell tumor	4 (33.3)	4 (33.3)	1 (8.3)	3 (25)	12
Germinoma	1 (16.6)	4 (66.6)	1 (16.6)	0 (0)	6
Choriocarcinoma	0 (0)	0 (0)	0 (0)	1 (100)	1

**Table 4:** Associations Between Relapse and Clinical Variables.

Variable	Total Patients (n)	Relapse n (%)	No Relapse n (%)	*p*-Value *
Sex				
Male	30	2 (6.7%)	28 (93.3%)	>0.05
Female	24	7 (29.2%)	17 (70.8%)	>0.05
Age group at diagnosis				
0–1 month	0	-	-	-
1–12 months	11	2 (18.2%)	9 (81.8%)	>0.05
12–60 months	22	5 (22.7%)	17 (77.3%)	>0.05
61–156 months	8	0 (0%)	8 (100%)	>0.05
157–216 months	13	2 (15.4%)	11 (84.6%)	>0.05
Tumor site				
Extragonadal	26	8 (30.8%)	18 (69.2%)	>0.05
Gonadal	28	1 (3.6%)	27 (96.4%)	>0.05
Histological subtype				
Endodermal sinus tumor	26	5 (19.2%)	21 (80.8%)	>0.05
Mixed germ cell tumor	12	4 (33.3%)	8 (66.7%)	>0.05
Others (mature/immature teratoma, germinoma, gonadoblastoma, etc.)	16	0 (0%)	16 (100%)	>0.05
Completeness of resection				
Total resection	40	6 (15%)	34 (85%)	>0.05
Partial resection	10	3 (30.0%)	7 (70.0%)	>0.05
No surgery/biopsy only	4	0 (0%)	4 (100%)	>0.05
AFP elevation at diagnosis				
AFP elevated	36	5 (13.9%)	31 (86.1%)	>0.05
AFP normal	18	4 (22.2%)	14 (77.8%)	>0.05
Metastasis at diagnosis				
Present	17	5 (29.4%)	12 (70.6%)	>0.05
Absent	37	4 (10.8%)	33 (89.2%)	>0.05
Radiotherapy				
Received	5	1 (20.0%)	4 (80.0%)	>0.05
Not received	49	8 (16.3%)	41 (83.7%)	>0.05
Disease stage				
Stage I	16	1 (6.2%)	15 (93.8%)	>0.05
Stage II	17	2 (11.8%)	15 (88.2%)	>0.05
Stage III	11	2 (18.2%)	9 (81.8%)	>0.05
Stage IV	10	3 (30.0%)	7 (70.0%)	>0.05

* Statistically significant difference, *p* < 0.05.

## Data Availability

The data presented in this study are available on request from the corresponding author. The data are not publicly available due to privacy and ethical reasons.

## Abbreviations

The following abbreviations are used in this manuscript:

GCT	Germ cell tumors
CNS	Central nervous system
AFP	Alpha-fetoprotein
hCG	Human chorionic gonadotropin
VAC	Vincristine, actinomycin-D, cyclophosphamide
BEP	Bleomycin, etoposide, cisplatin
ICE	Ifosfamide, carboplatin, etoposide)
VIP	Etoposide, ifosfamide, cisplatin
JEB	Carboplatin, etoposide, bleomycin

## References

[1] Mosbech C.H., Rechnitzer C., Brok J.S., Meyts E.R.-D., Hoei-Hansen C.E. (2014). Recent advances in understanding the etiology and pathogenesis of Pediatric Germ Cell Tumors. J. Pediatr. Hematol. Oncol..

[2] Schraw J.M., Sok P., Desrosiers T.A., Janitz A.E., Langlois P.H., Canfield M.A., Frazier A.L., Plon S.E., Lupo P.J., Poynter J.N. (2023). Associations between birth defects and childhood and adolescent germ cell tumors according to sex, histologic subtype, and site. Cancer.

[3] Ries L.A., Eisner M.P., Kosary C., Stinchcomb D.G., Howlader N., Horner M.J., Mariotto A., Miller B.A., Feuer E.J., Altekruse S.F. (2013). SEER Cancer Statistics Review, 1975–2005.

[4] Bhuta R., Shah R., Gell J.J., Poynter J.N., Bagrodia A., Dicken B.J., Pashankar F., Frazier A.L., Shaikh F. (2023). Children’s Oncology Group’s 2023 blueprint for research: Germ cell tumors. Pediatr. Blood Cancer.

[5] Monagel D.A., Tala A., Arwa A., Sereen B., Ilana H., Deena H., Omaima A., Naglla E. (2023). Clinical and pathological characteristics of extra-cranial germ cell tumors: A 30-year single-center experience in Saudi Arabia. Saudi Med. J..

[6] Ishiguro T., Tsuchida Y. (1994). Clinical significance of serum alpha-fetoprotein subfractionation in pediatric diseases. Acta Paediatr..

[7] Dehner L.P. (1983). Gonadal and extragonadal germ cell neoplasia of childhood. Hum. Pathol..

[8] Harms D., Jänig U. (1986). Germ cell tumours of childhood. Virchows Arch. A.

[9] Zambrano E., De Stefano D.V., Reyes-Múgica M. (2017). Pediatric germ cell tumors. Pathology and Biology of Human Germ Cell Tumors.

[10] Isaacs H. (2004). Perinatal (fetal and neonatal) germ cell tumors. J. Pediatr. Surg..

[11] Yoshida M., Matsuoka K., Nakazawa A., Inoue T., Kishimoto H., Nakayama M., Takaba E., Hamazaki M., Yokoyama S., Horie H. (2013). Sacrococcygeal yolk sac tumor developing after teratoma: A clinicopathological study of pediatric sacrococcygeal germ cell tumors and a proposal of the pathogenesis of sacrococcygeal yolk sac tumors. J. Pediatr. Surg..

[12] Yang C., Wang S., Li C.-C., Zhang J., Kong X.-R., Ouyang J. (2010). Ovarian germ cell tumors in children: A 20-year retrospective study in a single institution. Eur. J. Gynaecol. Oncol..

[13] Schmidt P., Haas R.J., Göbel U., Calaminus G. (2001). Results of the German studies (MAHO) for treatment of testicular germ cell tumors in children: An update. Klin. Padiatr..

[14] Nguyen H.A., Bui N. (2014). Characteristics of extracranial germ cell tumours in children at national hospital of pediatrics, Hanoi, Vietnam. Pediatr. Blood Cancer.

[15] Schneider D.T., Calaminus G., Koch S., Teske C., Schmidt P., Haas R.J., Harms D., Göbel U. (2004). Epidemiologic analysis of 1442 children and adolescents registered in the German germ cell tumor protocols. Pediatr. Blood Cancer.

[16] Vaysse C., Delsol M., Carfagna L., Bouali O., Combelles S., Lemasson F., Le Mandat A., Castex M.-P., Pasquet M., Moscovici J. (2010). Ovarian germ cell tumors in children: Management, survival, and ovarian prognosis. A report of 75 cases. J. Pediatr. Surg..

[17] Harms D., Leuschner I. (1989). Abdominal, retroperitoneal, and sacrococcygeal tumors of the newborn and very young infant. Eur. J. Pediatr..

[18] Swamy R., Embleton N., Hale J. (2008). Sacrococcygeal teratoma over two decades: Birth prevalence, prenatal diagnosis, and clinical outcomes. Prenat. Diagn..

[19] Cianci M.C., Fusi G., Morini F., Severi E., Morabito A., Grimaldi C. (2025). Fetal treatment of sacrococcygeal teratoma: State of the art. Front. Pediatr..

[20] Ries L.A., Melbert D., Krapcho M., Mariotto A., Miller B.A., Feuer E.J., Clegg L., Horner M.J., Howlader N., Eisner M.P. (2007). SEER Cancer Statistics Review, 1975–2004.

[21] Mann J.R., Raafat F., Robinson K., Imeson J., Gornall P., Sokal M., Gray E., McKeever P., Hale J., Bailey S. (2000). The United Kingdom Children’s Cancer Study Group’s second germ cell tumor study: Carboplatin, etoposide, and bleomycin are effective treatment for children with malignant extracranial germ cell tumors, with acceptable toxicity. J. Clin. Oncol..

[22] Cornejo K., Frazier M., Lee L., Kozakewich H.P.W., Young R.H. (2015). Yolk Sac Tumor of the Testis in Infants and Children: A Clinicopathologic Analysis of 33 Cases. Am. J. Surg. Pathol..

[23] De Backer A., Madern G.C., Oosterhuis J.W., Hakvoort-Cammel F.G., Hazebroek F.W. (2006). Ovarian germ cell tumors in children: A clinical study of 66 patients. Pediatr. Blood Cancer.

[24] Billmire D., Vinocur C., Rescorla F., Cushing B., London W., Schlatter M., Davis M., Giller R., Lauer S., Olson T. (2004). Outcome and staging evaluation in malignant germ cell tumors of the ovary in children and adolescents: An intergroup study. J. Pediatr. Surg..

[25] Ramanathan S., Prasad M., Vora T., Parambil B.C., Kembhavi S., Ramadwar M., Khanna N., Laskar S., Kurkure P., Qureshi S. (2022). Outcomes and prognostic variables of extracranial germ cell tumors in children and adolescents treated over a decade: A developing world perspective. Pediatr. Blood Cancer.

[26] Popadiuk S., Korzon M., Szumera M., Chybicka A., Szmyd K., Dzierzega M., Kowalczyk J.R., Wiśniewska-Slusarz H., Trelińska J., Wozniak W. (2003). Malignant germ cell tumors: Multicenter prospective trial in Polish Pediatric Group for Solid Tumors (years 1998–2000). Prz. Lek..

[27] Suita S., Shono K., Tajiri T., Takamatsu T., Mizote H., Nagasaki A., Inomata Y., Hara T., Okamura J., Miyazaki S. (2002). Malignant germ cell tumors: Clinical characteristics, treatment, and outcome. A report from the study group for pediatric solid malignant tumors in the Kyushu Area, Japan. J. Pediatr. Surg..

[28] LoCurto M., Lumina F., Alaggio R., Cecchetto G., Almasio P., Indolfi P., Siracusa F., Bagnulo S., De Bernardi B., De Laurentis T. (2003). Malignant germ cell tumors in childhood: Results of the first Italian cooperative study “TCG91”. Med. Pediatr. Oncol..

[29] Derikx J.P., De Backer A., Schoot L., Aronson D.C., de Langen Z.J., Hoonaard T.L.v.D., A Bax N.M., van der Staak F., van Heurn E.L.W. (2006). Factors associated with recurrence and metastasis in sacrococcygeal teratoma. Br. J. Surg..

[30] Yoneda A., Usui N., Taguchi T., Kitano Y., Sago H., Kanamori Y., Nakamura T., Nosaka S., Oba M.S. (2013). Impact of the histological type on the prognosis of patients with prenatally diagnosed sacrococcygeal teratomas: The results of a nationwide Japanese survey. Pediatr. Surg. Int..

[31] Frazier A.L., Hale J.P., Rodriguez-Galindo C., Dang H., Olson T., Murray M.J., Amatruda J.F., Thornton C., Arul G.S., Billmire D. (2014). Revised risk classification for pediatric extracranial germ cell tumors based on 25 years of clinical trial data from the United Kingdom and United States. J. Clin. Oncol..

